# A Critical Outlook for the Pursuit of Lower Contact Resistance in Organic Transistors

**DOI:** 10.1002/adma.202104075

**Published:** 2021-10-07

**Authors:** James W. Borchert, R. Thomas Weitz, Sabine Ludwigs, Hagen Klauk

**Affiliations:** ^1^ 1st Institute of Physics Georg August University of Göttingen Friedrich‐Hund‐Platz 1 37077 Göttingen Germany; ^2^ IPOC ‐ Functional Polymers Institute of Polymer Chemistry University of Stuttgart Pfaffenwaldring 55 70569 Stuttgart Germany; ^3^ Max Planck Institute for Solid State Research Heisenbergstraße 1 70569 Stuttgart Germany

**Keywords:** charge injection, contact resistance, Fermi‐level pinning, interfaces, organic transistors

## Abstract

To take full advantage of recent and anticipated improvements in the performance of organic semiconductors employed in organic transistors, the high contact resistance arising at the interfaces between the organic semiconductor and the source and drain contacts must be reduced significantly. To date, only a small portion of the accumulated research on organic thin‐film transistors (TFTs) has reported channel‐width‐normalized contact resistances below 100 Ωcm, well above what is regularly demonstrated in transistors based on inorganic semiconductors. A closer look at these cases and the relevant literature strongly suggests that the most significant factor leading to the lowest contact resistances in organic TFTs so far has been the control of the thin‐film morphology of the organic semiconductor. By contrast, approaches aimed at increasing the charge‐carrier density and/or reducing the intrinsic Schottky barrier height have so far played a relatively minor role in achieving the lowest contact resistances. Herein, the possible explanations for these observations are explored, including the prevalence of Fermi‐level pinning and the difficulties in forming optimized interfaces with organic semiconductors. An overview of the research on these topics is provided, and potential device‐engineering solutions are discussed based on recent advancements in the theoretical and experimental work on both organic and inorganic semiconductors.

## Graphical abstract

Though organic transistors have long been considered as a potential cornerstone technology for flexible electronics applications, their widespread commercialization in high‐frequency circuits has been substantially impeded by high contact resistance. A review of the state‐of‐the‐art reveals the pivotal role of the organic‐semiconductor morphology and the key challenges now to be overcome, such as Fermi‐level pinning and low charge‐carrier densities.

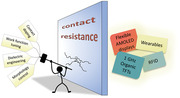

## 1 Introduction

Organic semiconductors have been implemented in a variety of electronic devices, including organic light‐emitting diodes (OLEDs),^[^ ^1^ ^]^ organic solar cells,^[^ ^2^ ^]^ organic photodetectors,^[^ ^3^ ^]^ and organic transistors of various forms.^[^ ^4^, ^5^, ^6^, ^7^ ^]^ An essential requirement for all these devices is the efficient injection and/or extraction of charges across the interfaces between the organic semiconductor(s) and the electrical contacts.^[^ ^8^ ^]^ Extensive research and development efforts into the active materials and the device engineering required for achieving efficient charge injection/extraction have therefore been instrumental in enabling, for instance, the commercialization of OLEDs. Progress in this area has now proceeded to the extent that charge injection and extraction are not the most critical limiting issues for the state‐of‐the‐art of OLEDs compared to other aspects, such as effective carrier and exciton confinement, energy transfer, outcoupling, and lifetime.^[^ ^9^, ^10^, ^11^, ^12^ ^]^ Much the same is true for organic solar cells, in that the primary focus and source of improvements recently have been more‐closely linked to the development of nonfullerene acceptors.^[^ ^13^ ^]^ Organic transistors of all types, on the other hand, which have been touted as a leading alternative to transistors based on inorganic semiconductors for novel large‐area integrated‐circuit applications for many years,^[^ ^14^, ^15^ ^]^ have yet to attain widespread adoption in consumer‐electronics applications.

Several of the disadvantages of organic transistors compared to inorganic transistors, such as generally lower charge‐carrier mobilities, poorer device uniformity, and reduced reliability,^[^ ^16^ ^]^ have seen significant improvements over time, such that a few commercially available devices utilizing organic thin‐film transistors (TFTs) are now becoming available.^[^ ^17^ ^]^ However, the contact resistance (*R*_C_) persists as a major impediment toward further development of circuits based on organic transistors.^[^ ^18^, ^19^, ^20^, ^21^ ^]^ This is especially true for the development of organic TFTs for low‐power, high‐frequency applications, such as mobile active‐matrix displays, since a high *R*_C_ limits the maximum unity‐current‐gain‐cutoff (transit) frequency that would otherwise be expected through device miniaturization.^[^ ^22^ ^]^ High contact resistance in organic TFTs continues to be a major problem despite significant strides in expanding both the breadth of knowledge of the physicochemical properties of metal–organic semiconductor interfaces^[^ ^23^, ^24^ ^]^ and the development of a diverse selection of material‐engineering methods for tailoring the contact interface to enhance charge injection/extraction in organic semiconductors.^[^ ^20^, ^25^, ^26^ ^]^ Indeed, the vast majority of the reported methods consider a “successful” method to be one that can reduce exceptionally high channel‐width‐normalized contact resistance (*R*_C_ *W*) often exceeding 100 kΩcm down to 100 Ωcm at best,^[^ ^20^ ^]^ which is still generally unacceptable for mobile applications that rely on lithium ion batteries.^[^ ^22^ ^]^ To date, only a handful of reports showing *R*_C_ *W* of less than 100 Ωcm in organic field‐effect transistors, including TFTs and electrolyte‐gated organic field‐effect transistors (EGOFETs),^[^ ^6^ ^]^ have been published, most of which having come only in the last half decade (**Table** **1**).^[^ ^27^, ^28^, ^29^, ^30^, ^31^, ^32^, ^33^, ^34^, ^35^, ^36^, ^37^ ^]^ Put simply, there are many ways to reduce very high contact resistance in organic field‐effect transistors, but it is evidently much harder to improve upon already “low” contact resistances of around 100 Ωcm, especially in TFTs.

**Table 1:** Reports of organic field‐effect transistors with channel‐width‐normalized contact resistance (*R*_C_ *W*) of less than 100 Ωcm

Refs. (Year)	Semiconductor (morphology)	Transistor architecture	Concept stated to be critical for low *R*_C_ *W*	*R*_C_ *W* [Ωcm]	2D charge‐carrier density [10^13^ cm^−2^]^a)^
^[^ ^27^ ^]^ (2007)	Pentacene (polycrystalline)	Coplanar	UV/ozone‐treated Au	80	0.5
^[^ ^28^ ^]^ (2010)	P3HT (polycrystalline)	Staggered	High charge‐carrier density from electrolyte gate insulator	1	20
	F8T2 (polycrystalline)	Staggered		14	20
^[^ ^29^ ^]^ (2013)	DNTT (polycrystalline)	Staggered	Contact doping and minimized semiconductor film thickness	80	0.5
^[^ ^30^ ^]^ (2017)	C_10_‐DNTT (polycrystalline)	Coplanar	Chemisorbed monolayer treatment of Au contacts and annealing in N_2_	75	0.1
^[^ ^31^ ^]^ (2018)	C_8_‐DNBDT‐NW (bilayer crystalline)	Staggered	Contact doping and low‐dimensional semiconductor film	47	0.6
^[^ ^32^ ^]^ (2019)	PDPP (polycrystalline)	Staggered	High charge‐carrier density from electrolyte gate insulator	2.7	3
^[^ ^33^ ^]^ (2019)	DPh‐DNTT (polycrystalline)	Staggered	Au contacts only	56	1.0
	DPh‐DNTT (polycrystalline)	Coplanar	Chemisorbed monolayer treatment of Au contacts and thin gate insulator	29	1.0
^[^ ^34^ ^]^ (2020)	DPh‐DNTT (polycrystalline)	Coplanar	Chemisorbed monolayer treatment of Au contacts and thin gate insulator	10	0.8
	C_10_‐DNTT (polycrystalline)	Coplanar		31	0.8
^[^ ^35^ ^]^ (2020)	C_10_‐DNTT (monolayer crystalline)	Staggered	Transferred Au contacts and low‐dimensional semiconductor film	40	0.9
^[^ ^36^, ^37^ ^]^ (2020)	C_9_‐DNBDT‐NW (bilayer crystalline)	Staggered	Contact doping and low‐dimensional semiconductor film	60, 50	0.4, 0.3

^a)^ If not explicitly reported, estimated based on the maximum drain current, channel width, and a channel “thickness” of 5 nm or the gate overdrive voltage and gate‐dielectric capacitance.

It is important to appreciate that even the best metrics of 10 Ωcm in organic TFTs^[^ ^34^ ^]^ and 1 Ωcm in EGOFETs^[^ ^28^ ^]^ are orders of magnitude higher than what is achievable in nearly all transistors based on inorganic semiconductors. The closest comparison that can be made is to state‐of‐the‐art indium gallium zinc oxide (IGZO) TFTs, which often show *R*_C_ *W* of around 10 Ωcm^[^ ^38^ ^]^ and can reach as low as 0.8 Ωcm.^[^ ^39^ ^]^ The difference is even more pronounced for transistors based on other classes of semiconductors. In fact, in transistors based on highly crystalline inorganic semiconductors, the contact resistance can be reduced nearly to the quantum limit determined by the 2D charge‐carrier density accumulated in the charge‐carrier channel of the transistor.^[^ ^40^, ^41^, ^42^ ^]^ This corresponds to around 0.01 Ωcm in both silicon metal–oxide–semiconductor field‐effect transistors (MOSFETs)^[^ ^43^ ^]^ and gallium nitride high‐electron‐mobility transistors (HEMTs).^[^ ^44^ ^]^ 2D semiconductors, of which transition‐metal dichalcogenides (TMDs) are of particular interest for some of the same application spaces as organic semiconductors and IGZO, typically achieve *R*_C_ *W* < 1 Ωcm these days,^[^ ^40^, ^41^, ^42^ ^]^ including a recent demonstration of 0.012 Ωcm in MoS_2_ transistors.^[^ ^42^ ^]^

It is thus no surprise that, due to the inexorable link between contact resistance and dynamic performance, even state‐of‐the‐art low‐voltage organic transistors still struggle to achieve a transit frequency on the order of 10 MHz,^[^ ^22^, ^31^, ^34^, ^36^, ^37^, ^45^, ^46^, ^47^ ^]^ with only a single recent result of over 100 MHz that notably required relatively high operating voltages, in part to combat the high contact resistance.^[^ ^48^ ^]^ In stark contrast, TMD and IGZO TFTs are able to already achieve maximum transit frequencies exceeding 1 GHz.^[^ ^49^, ^50^ ^]^ While the charge‐carrier mobility is also typically higher in TMDs and IGZO, it is worth noting that a transit frequency of 1 GHz was demonstrated in IGZO TFTs with an effective carrier mobility of only 1.2 cm^2^ V^−1^ s^−1^ and an intrinsic channel mobility of 18.2 cm^2^ V^−1^ s^−1^,^[^ ^45^ ^]^ similar to what is achievable in some premier organic semiconductors.^[^ ^18^, ^51^ ^]^ While a transit frequency of 10 MHz is sufficient for some targeted applications, such as low‐end flexible mobile displays,^[^ ^52^ ^]^ broadening the range of possible applications for organic transistors clearly requires significant further reduction of the contact resistance. However, this goal is likely only to be achieved through more‐focused efforts toward understanding the limiting factors in organic transistors that already show “low” contact resistances.

Here, a focused critical analysis of the various approaches that have yielded *R*_C_ *W* < 100 Ωcm in organic transistors is presented, with further discussion of the prospects that any of these given methods will enable further improvements beyond the state‐of‐the‐art, which as of writing stands at 10 Ωcm for high‐frequency organic TFTs.^[^ ^34^ ^]^ A necessary brief summary of the physical origins of the contact resistance is provided to facilitate a meaningful discussion of the merits of the discussed methods for reducing the contact resistance and about potential further routes toward improvement. The primary goal of this report is to clarify the important factors and mechanisms behind the methods that have led to the lowest contact resistances in organic transistors. For a more in‐depth review of the physics of charge injection in organic transistors and modeling of the contact resistance, the interested reader is referred to other recent and comprehensive reviews.^[^ ^20^, ^25^, ^26^, ^53^, ^54^ ^]^ After this more general overview, focused discussions are provided on the potentially augmented role of Fermi‐level pinning (FLP)^[^ ^55^, ^56^ ^]^ at the metal–organic semiconductor interfaces in organic transistors with low contact resistance, since this imposes potentially severe limitations on the capabilities of any method aimed at reducing the injection‐barrier height. Further discussion is provided on methods to combat FLP, including the role of the organic semiconductor itself^[^ ^56^, ^57^, ^58^ ^]^ and the difficulties in forming more idealized interfaces between the contacts and the organic semiconductor,^[^ ^35^, ^59^, ^60^, ^61^ ^]^ as these are potentially the key parameters for enabling further reduction of the contact resistance in state‐of‐the‐art high‐frequency organic TFTs. Consideration will also be given to the extent to which methods for improving the contact resistance are applicable to the miniaturization of organic transistors to nanoscale dimensions, which remains the essential route toward enhancing the dynamic performance of integrated circuits based on organic transistors.^[^ ^22^, ^62^, ^63^ ^]^ The discussion will include key results and ideas from the literature on these topics, including potential insights from outside the organic‐electronics community, especially with regards to contacts to 2D semiconductors like TMDs.^[^ ^41^ ^]^

## 2 Contact Resistance in Organic Transistors

In this section, we will give a brief overview of the most important aspects of the contact resistance in organic transistors.

### 2.1 Critical Impacts of the Device Architecture

**Figure 1:**
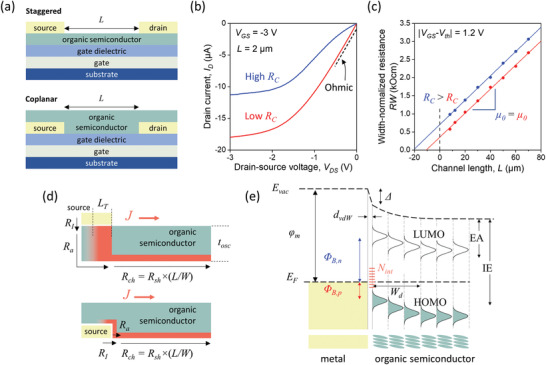
a) Schematic cross‐sections of the two main device architectures utilized for organic transistors. The source and drain contacts are separated by a channel of length *L*. b) Output curves of two organic transistors with a relatively short channel of *L* = 2 µm. The blue curve shows evidence of a high, non‐Ohmic contact resistance compared to the red curve. c) Gated transmission line method (TLM) analysis corresponding to the transistors in panel (b) showing that they are identical other than in terms of the width‐normalized contact resistance (*R*_C_ *W*). d) Schematic diagram of the current density (*J*) in the semiconductor near the source contact in staggered and coplanar transistors, separating the contributions to the total resistance (*R*) into the interface (*R*_I_) and access (*R*_A_) resistance components of the contact resistance (*R*_C_) and the channel resistance (*R*_ch_). e) Simplified schematic energy diagram of the interface between a metal and an organic‐semiconductor film in thermal equilibrium. The diagram depicts the situation in a p‐channel transistor without applied voltages. φ_m_: work function of the metal. Δ: interface dipole. Φ_B,n_: energy barrier for electrons. Φ_B,n_: energy barrier for holes. *N*_int_: interface states. *d*_vdW_: width of the van der Waals gap. *W*_d_: width of the depletion region. EA: electron affinity. IE: ionization energy. e) Adapted with permission.^[^ ^58^ ^]^ Copyright 2017, IOP Publishing.

There are many intrinsic and extrinsic factors determining the origins of the contact resistance in organic transistors. Potentially, the most impactful and widely studied extrinsic factor in organic TFTs is the choice of the device architecture.^[^ ^64^, ^65^, ^66^, ^67^, ^68^, ^69^ ^]^ The different device architectures for organic TFTs can generally be grouped into coplanar and staggered configurations (**Figure** **1**a). This distinction refers simply to the position of the source and drain contacts with respect to the interface between the organic semiconductor and the gate dielectric. Both architectures have shown advantages and disadvantages in terms of the impacts on various performance characteristics of the TFTs, such as gate coupling and charge injection.^[^ ^66^, ^68^, ^69^, ^70^ ^]^ The conventional wisdom is that staggered TFTs should have smaller contact resistance than coplanar TFTs, based on experimental data from organic TFTs comprising the same materials and film thicknesses,^[^ ^64^, ^67^, ^71^, ^72^, ^73^, ^74^ ^]^ and from theoretical device simulations and physical models.^[^ ^66^, ^67^, ^73^, ^74^ ^]^ The standard argument is that the effective area for charge injection is larger and hence the contact resistance is smaller in staggered TFTs than in coplanar TFTs.^[^ ^67^, ^75^, ^76^ ^]^ However, there are other important factors for consideration that make a firm statement about which architecture is more advantageous difficult to completely justify.

The charge‐injection efficiency in staggered TFTs is highly sensitive to the thickness of the organic‐semiconductor layer due to the direct effects that this has on the access resistance.^[^ ^29^, ^75^, ^77^ ^]^ In addition, a gate‐voltage dependence of the contact resistance arises due to screening of the gate field by charges accumulated in the semiconductor between the source contact and the gate.^[^ ^70^ ^]^ In the case of top‐contact (TC) staggered TFTs, the semiconductor morphology and the method by which the contacts are deposited can also have significant impacts on the metal–organic semiconductor interface and on the efficiency of charge transport away from the contact region.^[^ ^35^, ^59^, ^78^, ^79^ ^]^ Likewise, in bottom‐contact (BC) coplanar TFTs, any nonidealities in the semiconductor morphology on the contact surface and along the edges of the contacts may drastically degrade the charge‐injection efficiency.^[^ ^60^, ^80^, ^81^ ^]^ Coplanar contacts have been shown to provide low contact resistance, so long as the semiconductor morphology is sufficiently controlled at the interfaces between the contacts, the gate dielectric and the organic semiconductor.^[^ ^27^, ^81^, ^82^ ^]^ Moreover, it was recently demonstrated that when a very thin gate dielectric is implemented in coplanar TFTs, the contact resistance can be even lower than in comparable staggered TFTs.^[^ ^33^, ^83^ ^]^ Due to these and other complex competing factors that can arise in the fabrication of organic transistors, the historical record on which device architecture is capable of showing the lowest contact resistance in organic transistors is somewhat ambiguous.^[^ ^34^ ^]^ Suffice to say, at this point, the differences in the contact resistances between sufficiently comparable state‐of‐the‐art coplanar and staggered organic TFTs are relatively small, as will become clear in this report.

### 2.2 Discerning “High” and “Low” Contact Resistance

Regardless of the specific architecture or other potentially contributing extrinsic issues from fabrication, the contact resistance is generally identified by the observation that a significant portion of the drain–source voltage (*V*_DS_) drops across the contacts, rather than along the channel region of the transistor.^[^ ^84^, ^85^, ^86^ ^]^ This parasitic voltage drop (*V*_C_) tends to be non‐Ohmic in organic transistors with significant dependence on the applied electric fields^[^ ^26^, ^76^, ^86^, ^87^, ^88^ ^]^ but can attain Ohmic‐like character under certain conditions.^[^ ^75^ ^]^ Injection can additionally be influenced by the mobility and the density of the charge‐carriers in the organic semiconductor near to the contact interface, especially in the case of highly disordered semiconductors dominated by hopping (diffusion‐limited) transport.^[^ ^89^, ^90^, ^91^, ^92^ ^]^ Furthermore, there is evidence to suggest that the carrier mobility in the channel region also can have a direct impact on the contact resistance, though the precise nature of this influence and how it should be modeled is still under active investigation.^[^ ^54^, ^73^, ^87^, ^92^, ^93^ ^]^

Generally speaking, in transistors with sufficiently long channel lengths such that the greatest portion of the total resistance (*R*) is determined by the sheet resistance of the semiconductor layer (*R*_sh_), the current–voltage characteristics of the transistors can give the impression of “Ohmic” injection, in which case the contact resistance is usually considered “low.” Ohmic contact resistance is achieved when the voltage drop across the contacts is negligible compared to the total voltage drop in the device.^[^ ^26^, ^84^ ^]^ Truly “Ohmic” contact resistance is only accomplished in organic transistors when *V*_DS_ is sufficiently low that fewer charge carriers per unit volume are injected than are present in the bulk via thermal activation or application of a gate–source voltage (*V*_GS_). Owing to the low permittivity of organic semiconductors, as *V*_DS_ and *V*_GS_ are increased, the Schottky barrier is reshaped and reduced in height by the Coulombic interactions between the injected charges in the semiconductor and the corresponding image charges on the metal contact.^[^ ^53^, ^70^, ^89^, ^92^, ^94^ ^]^ The excess injected charges are diffusively transported away from the contacts according to space‐charge limitations in the organic semiconductor, and the current–voltage characteristics become nonlinear.^[^ ^95^, ^96^ ^]^ A non‐Ohmic (“high”) contact resistance can often be clearly observed in the output curves of transistors with sufficiently small channel length (*L*), where a distinctive “S” shape appears when the injection barriers are large (Figure 1b).^[^ ^70^ ^]^

For the rest of this report, the terms “low” and “high” for the contact resistance are used with a cutoff set at *R*_C_ *W* = 100 Ωcm, rather than as synonyms for Ohmic or non‐Ohmic contacts, respectively. Indeed, some state‐of‐the‐art organic TFTs with the lowest overall contact resistances can still show non‐Ohmic behaviors for sufficiently small channel lengths, such as field dependence and barrier‐lowering effects.^[^ ^87^, ^97^ ^]^

### 2.3 Measurement of the Contact Resistance

The assumption of an Ohmic contact resistance along with adherence to the gradual channel approximation (GCA) in the channel of the TFT^[^ ^98^ ^]^ allows the contact resistance to be measured using the gated transmission line method (TLM), wherein the transfer characteristics of transistors with different channel lengths are measured at a low drain–source voltage (*V*_DS_), i.e., in the linear regime of operation.^[^ ^99^ ^]^ In a TLM analysis, the source resistance and the drain resistance are lumped into a single term denoted as *R*_C_, which generally includes contributions from the interface resistance (*R*_I_) and the access resistance arising from the resistivity of the organic semiconductor (*R*_A_) (Figure 1d). Approaches to reduce the contact resistance are generally aimed at addressing one or more of these contributions, which are rooted in the physicochemical characteristics of the interface between the (typically) metal contacts and the organic semiconductor.^[^ ^100^ ^]^ Since the contact resistance is known to depend on the charge‐carrier density and field dependence of the mobility, the most common practice for reporting a single value for the contact resistance from a TLM analysis is to take the value extracted at the highest gate overdrive voltage (*V*_GS_–*V*_th_), where *V*_th_ is the threshold voltage (Figure 1c). In staggered transistors, a critical further quantity that can be estimated using TLM is the transfer length (*L*_T_) that, along with the channel width (*W*), determines the area over which the majority of charge‐carrier injection occurs.^[^ ^76^, ^101^ ^]^ In light of the discussion in the previous section, it should be noted that the key premise of the TLM, namely, that the contact resistance is Ohmic and independent of the channel length, does not necessarily hold, due to the fact that the contact resistance is inherently nonlinear and therefore does show a non‐negligible dependence on the channel length, even for relatively ideal TFTs.^[^ ^76^, ^87^, ^97^, ^102^ ^]^ Nevertheless, while single‐device characterization methods, such as the gated four‐probe (gFP) method^[^ ^65^, ^79^ ^]^ and Kelvin probe force microscopy (KPFM),^[^ ^85^, ^86^ ^]^ can provide more detailed and potentially more accurate descriptions of the potential profiles at the contacts, the TLM is by far the most common approach for determining the contact resistance of organic as well as inorganic TFTs, due to its simplicity of implementation and its advantages in terms of providing minimal statistics for several devices.^[^ ^99^ ^]^

There are a limited number of reliable approaches for the measurement of the contact resistance while the TFTs are operated in the saturation regime.^[^ ^103^ ^]^ It was recently shown that measurements of the transit frequency (*f*_T_) of TFTs with multiple channel lengths may also be used for this purpose.^[^ ^34^ ^]^

### 2.4 Physical Origins of the Contact Resistance in Organic Transistors

When two dissimilar materials are brought into physical contact, charge rearrangement spontaneously occurs across the interface to bring the combined system to thermal equilibrium, i.e., a common Fermi level (*E*_F_) is established.^[^ ^84^ ^]^ Figure 1e shows a simplified energy diagram for a high‐work function metal making contact to an organic semiconductor in a p‐channel transistor.^[^ ^58^ ^]^ The resulting equilibrium (re)arrangement of the energy levels and charge distributions across the interface between the metal contact and the semiconductor will then to a first degree determine the charge‐transport processes that dominate during device operation, which can be broadly grouped into thermally activated and quantum‐tunneling‐mediated transport of carriers across the interface.^[^ ^104^ ^]^ The most crucial aspects to consider for these charge injection/extraction processes between the metal and the organic semiconductor are the energy (Schottky) barriers (*Φ*_B_) that form at the interface, the width of the interfacial van der Waals gap (*d*_vdW_) between the metal contact and the first layer of the organic‐semiconductor film, the presence of an interface dipole (*Δ*), and the width of the depletion region (*W*_d_) determined by the energetic landscape and charge‐carrier density in the semiconductor.^[^ ^61^ ^]^ For the organic transistors with the lowest contact resistances, charge injection can generally be described well by thermionic‐emission theory for Schottky diodes in which the current density (*J*)follows^[^ ^25^, ^41^, ^84^ ^]^

(1) $$J=A^{*}T^{\alpha }\exp\left(\frac{-\Phi_{\;B}}{k_{B}T}\right)\left(1-\exp\left(-\frac{\Delta \Phi_{\;B}+qV_{DS}}{k_{B}T}\right)\right)$$

where A* is the effective Richardson constant, α is the dimensionality constant (equal to 2 for bulk semiconductors and 3/2 for 2D semiconductors), *q* is the elementary charge, and Δ*Φ*_B_ is the reduction in the Schottky barrier height due to image forces. For *V*_DS_ = 0 and α = 2, the contact resistance then follows

(2) $$R_{c}=\left(\frac{k_{B}}{qA^{*}T}\right)\;\exp\left(\frac{\Phi_{B}}{k_{B}T}\right)$$

The exponential dependence of the contact resistance on *Φ*_B_ means that this is in principle the most critical parameter for improving the contact resistance.^[^ ^25^, ^63^ ^]^ That being the case, the material parameter that has often been given the most attention is the work function of the contacts (φ_C_), defined as the difference between the vacuum energy level (*E*_vac_) and the Fermi energy (*E*_F_) of the isolated contact material. For a pure metal contact, this is taken to be the work function of the metal (φ_m_), and thus this is what is used in the simplified energy diagram in Figure 1e. Assuming vacuum‐level alignment, comparison of φ_m_ to the energies of the transport levels of the isolated semiconductor (ionization energy (IE) and electron affinity (EA)) then gives an initial guideline for the expected magnitude of the injection barrier for holes (*Φ*_B,p_) and electrons (*Φ*_B,n_). While this can serve as a starting point for contact engineering, this simplified view is essentially never entirely accurate, since multiple other extrinsic factors contribute to the final equilibrium state that is realized after contact formation (Figure 1e)_._^[^ ^23^, ^100^, ^105^, ^106^ ^]^ While this is generally true of all metal–semiconductor contacts, there are notable unique aspects that dominate the detailed energetic characteristics of metal–organic semiconductor interfaces that ultimately determine the charge‐injection physics.^[^ ^100^ ^]^

Perhaps the most well‐known and studied phenomenon in the research into the complex physics of metal–organic semiconductor interfaces is the common presence of a pronounced interface dipole (*Δ*),^[^ ^107^ ^]^ which can be quantified, e.g., by analyzing the distribution of the occupied states in the organic semiconductor across the semiconductor thickness by ultraviolet photoelectron spectroscopy (UPS).^[^ ^108^, ^109^ ^]^ The interface dipole has many noted possible origins, relating primarily to the localized energy levels in organic semiconductors.^[^ ^110^ ^]^ This includes charge transfer across the interface leading to the formation of a surface sheet charge on the metal with an associated image charge‐carrier density in the organic semiconductor, strong chemical reactions between the organic‐semiconductor molecules and ultraclean metal surfaces, variations in the orientation of the molecules at the surface and inside the semiconductor film, intrinsic dipole moments within the adsorbate molecules themselves, and the reduction of the metal work function by the interaction of the π‐electron clouds of a physisorbed organic layer with the metal electron tail states (referred to as the “pillow” or “push‐back” effect).^[^ ^26^, ^107^, ^108^, ^109^, ^110^, ^111^ ^]^ Furthermore, the redistribution and accumulation of charges in the organic semiconductor leads to band bending.^[^ ^58^ ^]^ Band bending is typically quantified by the extent over which the transport levels vary, proceeding from the metal surface into the organic semiconductor away from the contact interface (*W*_d_).^[^ ^92^, ^107^ ^]^ While it is common practice to represent the bending of the energy levels as continuous, this is not strictly speaking appropriate for organic semiconductors, due to the more localized nature of the molecular orbitals. This is especially true for small‐molecule semiconductors that tend to grow in a layer‐by‐layer mode,^[^ ^112^ ^]^ as shown schematically in Figure 1e.^[^ ^58^ ^]^ Finally, depending on the shape and distribution of the density of states (DOS) of the highest occupied molecular orbital (HOMO) and lowest unoccupied molecular orbital (LUMO) (represented by Gaussian distributions in Figure 1e) and the potential presence of interfacial mid‐gap states (*N*_it_) arising from disorder or impurities, the equilibrium position of the Fermi level with respect to the transport levels of the organic semiconductor may become “pinned,” such that changes in the contact work function do not affect the final barrier heights (*Φ*_B,p_, *Φ*_B,n_).^[^ ^23^, ^55^, ^56^, ^77^, ^110^, ^113^ ^]^ This effect is known as FLP and is discussed in greater detail in a latter section.

The further adherence to thermionic‐emission theory for charge‐carrier transport across the interface between the contact and the organic semiconductor depends on the charge‐carrier density and the efficiency of the charge‐carrier transport in the semiconductor layer.^[^ ^84^, ^91^, ^92^, ^114^ ^]^ A key aspect that determines the efficiency of charge‐carrier transport in organic semiconductors, as well as the quality of the contact–semiconductor interface, is that the molecules interact with each other and crystallize through weak van der Waals forces, rather than covalent bonds.^[^ ^112^ ^]^ The DOS of the HOMO and LUMO are thus strongly affected not only by the chemical structure, but also by both the arrangement of the molecules in the organic semiconductor and the concentration of any dopants that could potentially be introduced to change the charge‐carrier‐transport characteristics.^[^ ^57^, ^115^ ^]^ Efficient transport of charge in organic semiconductors relies in large part on the degree of overlap of the π‐orbitals between adjacent molecules, which can be drastically affected by any differences in the semiconductor morphology.^[^ ^51^, ^116^ ^]^ The mean free path of mobile charge carriers in organic semiconductors is thus typically very small compared to inorganic semiconductors (on the order of the molecular separation distances), and is highly sensitive to, e.g., thermal fluctuations and extrinsic factors, such as the surface roughness of the substrate and surface contaminants, leading to small transfer integrals and diffusion‐limited transport of charges.^[^ ^51^, ^91^, ^92^, ^115^ ^]^

In most cases, the real physical interfaces formed between the contacts and the organic‐semiconductor layer in a device are not as atomically sharp as can be achieved in inorganic‐semiconductor devices, but are rather ill‐defined and prone to increased disorder depending on the device‐fabrication approach that is implemented. Diffusion of metal nanoclusters and thermal damage to the organic semiconductor often occur in the case of metals deposited directly onto the organic‐semiconductor layer, such as in top‐contact TFTs.^[^ ^59^, ^79^ ^]^ On the other hand, amorphous or polycrystalline thin films tend to form when the organic semiconductor is deposited directly onto a bare metal contact due to large surface roughness and/or suboptimal control of the surface energy, which is typically different on the contacts than on the surface of the gate dielectric, such as in bottom‐contact TFTs.^[^ ^60^, ^81^, ^117^ ^]^ A short mean free path leads to diffusion‐limited hopping transport of the injected charge, resulting in a wide depletion region and subsequently increased dependence of the charge‐injection efficiency on the charge‐carrier mobility in the organic semiconductor close to the contacts due to space‐charge limitations.^[^ ^26^, ^92^, ^93^, ^118^ ^]^ This was shown to be particularly true of many amorphous semiconducting polymers where the mobility of the charge carriers tends to be low and recombination rates with image charges at the metal contacts are high.^[^ ^92^ ^]^ By contrast, inorganic semiconductors characterized by highly delocalized transport of charges in well‐defined energy bands can be described by thermionic emission rather well, since the mean free path of the carriers is large and contributions from diffusion‐limited transport are negligible.^[^ ^41^, ^84^ ^]^ In more‐crystalline organic‐semiconductor thin‐films, such as low‐dimensional liquid crystals,^[^ ^119^ ^]^ the charge‐carrier injection from the metal contacts can also follow thermionic emission quite closely,^[^ ^35^, ^120^, ^121^ ^]^ and the contact resistance in transistors based on such materials can be quite small.

In summary, the physical origins of the contact resistance in organic transistors are a complex combination of competing intrinsic and extrinsic factors, all of which have received some level of attention in the community in order to optimize charge‐carrier injection. However, as will be seen in the following critical overview of the lowest contact resistances so far demonstrated, because of the above‐outlined substantial effects that the thin‐film morphology of the organic semiconductor has on the local energetics and on charge‐carrier transport, controlling the morphology of the organic semiconductor close to the metal contacts is the most prevalent requirement. Indeed, while minimization of the height of the Schottky barrier, e.g., by tuning the contact work function, has received a large amount of the attention, it is evidently of secondary importance when attempting to achieve the most efficient carrier injection possible regardless of the organic TFT architecture.^[^ ^77^, ^80^ ^]^

## 3 Survey of Organic Transistors Showing *R*_C_ *W* < 100 Ωcm

In this section, the handful of reports of organic TFTs and EGOFETs that have shown channel‐width normalized contact resistances of less than 100 Ωcm (Table 1) are discussed in a mostly chronological fashion. It is worth highlighting that these contact resistances have been accomplished using various methods rather than one ubiquitous approach, each in some way affecting one or more aspects of the contact–organic semiconductor interface. With only one exception,^[^ ^34^ ^]^ the contact resistances in these reports were measured using the gated TLM.^[^ ^99^ ^]^

### 3.1 First Realization of the Critical Role of Semiconductor‐Morphology Control

**Figure 2:**
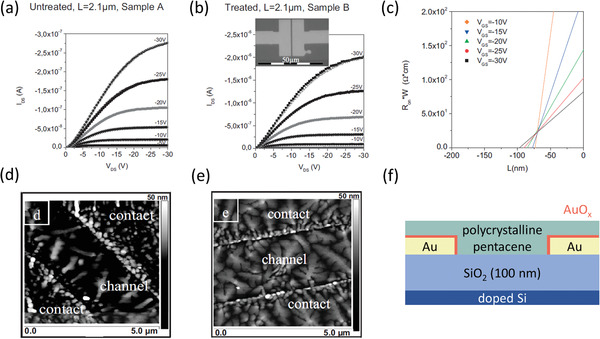
Output curves of bottom‐gate, bottom‐contact pentacene TFTs with a) untreated gold contacts and b) gold contacts exposed to UV/ozone prior to the pentacene deposition. c) TLM analysis of TFTs with UV/ozone‐treated contacts. d) AFM topographical scan of the channel region of a TFT with untreated contacts. e) AFM topographical scan of the channel region of a TFT with UV/ozone‐treated contacts. f) Schematic cross‐section of the TFTs with the UV/ozone‐treated contacts. Adapted with permission.^[^ ^27^ ^]^ Copyright 2007, John Wiley & Sons.

The first report of organic transistors with an *R*_C_ *W* of less than 100 Ωcm was published by Stadlober et al. in 2007 (**Figure** **2**).^[^ ^27^ ^]^ Here, a coplanar bottom‐gate, bottom‐contact architecture was implemented, using a 100 nm thick SiO_2_ gate dielectric, vacuum‐deposited pentacene as the active layer and gold source and drain contacts that were exposed to UV/ozone to produce a thin layer of gold oxide on the contact surface. As the title of the article states, the best result (*R*_C_ *W* = 80 Ωcm) was at the time (and remains still) orders of magnitude lower than in most organic transistors.^[^ ^34^ ^]^ This was a significant work for several other reasons. The channel lengths (*L*) that were implemented in this study were as small as 370 nm, by virtue of using nanoimprint lithography.^[^ ^122^ ^]^ This showed that the UV/ozone treatment for reducing the contact resistance is readily suitable for device scaling, which is essential for increasing the dynamic performance of organic transistors.^[^ ^22^ ^]^ To address the mechanisms behind the contact‐resistance improvement, Stadlober et al. noted that the gate‐independent contribution to the contact resistance (*R*_C,0_), determined by the convergence point of the linear fits in the TLM analysis of the width‐normalized total on‐state resistance (*R*_on_ *W*) at different *V*_GS_ as a function of *L* (Figure 2c),^[^ ^78^, ^99^ ^]^ was still relatively large (≈50 Ωcm) compared to the total contact resistance. Using the common interpretation for *R*_C,0_ to be representative of the interface resistance (*R*_I_) arising from the height of the Schottky barrier at the interface,^[^ ^77^, ^99^ ^]^ this implies that the deciding factor for the lower *R*_C_ compared to the transistors with untreated gold contacts was the improvement in the gate‐voltage‐dependent access resistance (*R*_A_) to the channel region through the pentacene layer by virtue of an improvement in the thin‐film morphology both on the treated contacts and in the channel region directly next to the contacts (Figure 2d,e). This provided one of the first pieces of experimental evidence that the strong influence of the semiconductor morphology at the interface with the contacts on charge exchange is of vital importance for reducing *R*_C_ *W* below 100 Ωcm, perhaps even exceeding the importance of reducing the nominal Schottky barrier height.

### 3.2 Ultrahigh Charge‐Carrier Density in Electrolyte‐Gated Organic Transistors

**Figure 3:**
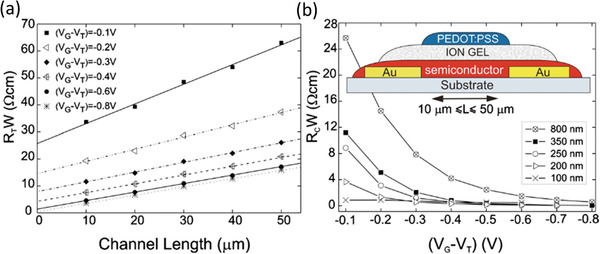
The “ultralow” contact resistance of electrolyte‐gated organic transistors is achieved by penetration of the ions from the ion‐gel electrolyte into the organic‐semiconductor layer near the contacts. a) TLM analysis. b) Results from TLM for different thicknesses of the P3HT layer. Adapted with permission.^[^ ^28^ ^]^ Copyright 2010, American Institute of Physics.

The results from Stadlober et al.^[^ ^27^ ^]^ unfortunately did not lead to such low contact resistances becoming immediately common‐place in organic TFTs. Indeed, despite a variety of new methods developed in the following decade,^[^ ^20^, ^25^ ^]^ only three additional reports of *R*_C_ *W* < 100 Ωcm were published.^[^ ^28^, ^29^, ^30^ ^]^ It is also intriguing that in these three cases, three entirely different methods were implemented, to the notable exclusion of the already proven benefit of UV/ozone treatment of the contacts. In 2010, Braga et al. reported on EGOFETs based on poly(3‐hexylthiophene) (P3HT) and poly(9,9‐dioctylfluorene‐*co*‐bithiophene) (F8T2) that had exceptionally low contact resistances (as low as *R*_C_ *W* = 1 Ωcm for P3HT; shown in **Figure** **3**).^[^ ^28^ ^]^ These impressively small contact resistances were achieved by virtue of extremely high charge‐carrier densities induced in the semiconductor film, with an estimated 2D carrier density of 2 × 10^14^ cm^−2^. This is afforded by the ion‐gel electrolyte employed as the gate insulator (Figure 3b), which forms an electric double layer with high electric field strength^[^ ^123^, ^124^ ^]^ and from which ions can diffuse into the organic semiconductor under the influence of the applied electric field.^[^ ^124^ ^]^ This importantly includes the semiconductor film in the region near to the contact interface, and is thought to reduce the width of the depletion region, significantly reducing the access resistance. More recently, Lenz et al. demonstrated that this operating principle can be applied to ultrashort channel, vertical organic field‐effect transistors (VOFETs), with *R*_C_ *W* = 2.7 Ωcm measured in otherwise similar lateral EGOFETs.^[^ ^32^ ^]^

Ion diffusion into the semiconductor is also a key operating principle behind organic electrochemical transistors (OECTs)^[^ ^125^ ^]^ that show in some cases even lower contact resistances than EGOFETs, owing to mixed ion and electron conduction.^[^ ^126^, ^127^ ^]^ As of the writing of this report, EGOFETs and OECTs stand as the overall benchmark to beat for organic transistors in terms of the contact resistance. It is worth noting that the current record standing at 0.5 Ωcm for OECTs was based on crystallized poly(3,4‐ethylenedioxythiophene) polystyrene sulfonate (PEDOT:PSS), highlighting again the importance of the organic semiconductor morphology on the contact resistance.^[^ ^127^ ^]^ EGOFETS and OECTs also include the only examples of *R*_C_ *W* < 100 Ωcm yet reported for transistors based on polymer semiconductors. Unfortunately, EGOFETs and OECTs are limited in their suitability for high‐frequency applications, since switching between the “on” and “off” states of the transistor is achieved in the case of EGOFETs through the movement of ions in the electrolyte and in the case of OECTs through electrochemical doping of the semiconductor, both of which are diffusion‐limited processes that are inherently slow compared to promoting channel formation in a field‐effect transistor.^[^ ^6^, ^125^ ^]^ It is additionally unclear whether the high charge‐carrier densities achievable in EGOFETs and OECTs can ever be realized in organic TFTs through, e.g., molecular doping (see following section). Nonetheless, these results provide some motivational proof that organic TFTs based on conventional (i.e., nonelectrolyte) gate dielectrics could possibly attain contact resistances on the order of those based on some inorganic semiconductors, like IGZO,^[^ ^39^ ^]^ if the charge‐carrier density can be sufficiently enhanced in semiconductor region close to the contacts.

### 3.3 Contact Doping and Reduction of the Semiconductor Thickness

**Figure 4:**
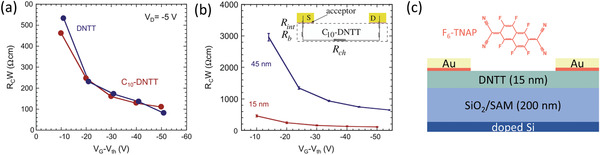
Results of TLM analysis of bottom‐gate, top‐contact organic TFTs utilizing contact doping. The small‐molecule acceptor F_6_‐TNAP (also referred to as F_6_‐TCNNQ) was used to dope the contact regions to enhance the hole transport in thin films of the small‐molecule semiconductors DNTT and C_10_‐DNTT. a) *R*_C_ *W* as a function of the gate overdrive voltage. b) Demonstration of the influence of the thickness of the organic‐semiconductor layer on the contact resistance. Adapted with permission.^[29]^ Copyright 2013, Elsevier. c) Schematic cross‐section of the TFTs.

Rather than ionic doping from an electrolyte, contact doping with ionic or organic molecules in the organic semiconductor host presents a potential alternative approach to achieve higher charge‐carrier densities in organic semiconductors^[^ ^128^, ^129^, ^130^, ^131^ ^]^ and has also seen a measure of success in reducing the contact resistance in nanoscale organic TFTs^[^ ^109^ ^]^ and in organic permeable‐base transistors (OPBTs).^[^ ^47^ ^]^ In 2013, Matsumoto et al. demonstrated *R*_C_ *W* < 100 Ωcm using a small‐molecule dopant to modify the contact properties of the TFTs (**Figure** **4**).^[^ ^29^ ^]^ There, the acceptor 1,3,4,5,7,8‐hexafluorotetracyanonaphthaquinodimethane (F_6_‐TNAP),^[^ ^133^ ^]^ which has a deep LUMO level of 5.37 eV, was used to dope the contact regions of bottom‐gate, top‐contact TFTs based on vacuum‐deposited dinaphtho[2,3‐*b*:2′,3′‐*f*]thieno[3,2‐*b*]thiophene (DNTT) and 2,9‐didecyldinaphtho[2,3‐*b*:2′,3′‐*f*]thieno[3,2‐*b*]thiophene (C_10_‐DNTT).^[^ ^134^ ^]^ The potentially most crucial finding in this work was the demonstration of the critical importance of minimizing the thickness of the organic semiconductor to reduce the access resistance in staggered devices (Figure 4b). Indeed, it is evident that reducing the thickness of the DNTT layers to 15 nm was instrumental in achieving *R*_C_ *W* < 100 Ωcm (Figure 3b). This report is also notable in that it was the first demonstration of the potential for low contact resistance in organic TFTs based on DNTT and its functional derivatives, which unsurprisingly also show among the highest hole mobilities out of all organic semiconductors.^[^ ^51^, ^135^, ^136^, ^137^ ^]^ Combining these factors resulted in a lowest *R*_C_ *W* of ≈80 Ωcm in their report (Figure 4a).

### 3.4 Chemical Modifications to the Contact Surfaces and Annealing

Rather than increasing the charge‐carrier density through contact doping, the contact resistance can also be reduced in principle by the insertion of various interlayers between the contacts and the organic‐semiconductor layer, with the intention of enabling more efficient injection from the metal to the transport levels by changing the contact work function or by introducing intermediate states to assist in charge transport.^[^ ^138^, ^139^, ^140^, ^141^ ^]^ In the case of bottom‐contact device architectures, this can take the form of either physisorbed or chemisorbed species. A particularly common and versatile method that is primarily used to improve the organic‐semiconductor morphology and reduce the trap‐state density on different surfaces is to modify the surface energy and reactivity by forming a self‐assembled monolayer (SAM) on the surface.^[^ ^142^ ^]^ SAMs have been an especially effective tool for tuning the surfaces of both the gate dielectric and the contacts in organic TFTs to improve the charge‐transport characteristics of the organic‐semiconductor layer.^[^ ^143^, ^144^, ^145^ ^]^ On noble‐metal contacts, such as gold or silver, molecules with a thiol (—SH) anchoring group are used to selectively bind to the surface of the contacts through oxidative addition of the S—H bond to the metal surface, followed by a reductive elimination of the hydrogen.^[^ ^60^, ^74^, ^81^, ^117^, ^141^, ^146^, ^147^, ^148^, ^149^, ^150^, ^151^, ^152^, ^153^, ^154^, ^155^, ^156^, ^157^, ^158^ ^]^ These thiol‐based SAMs can be used to modify the work function of the metal contacts, with the magnitude of the energy shift depending on the collective dipole moment of the SAM that is controlled by the structural chemistry of the molecules to be assembled on the surface, such as by simply changing the position of a polar group relative to the thiol anchor.^[^ ^151^, ^159^ ^]^ The dipolar character and the influence of a SAM can be beneficial for lowering the contact resistance in organic TFTs by tuning the work function by up to a few hundred millielectronvolts, bringing it closer to the desired transport level of the semiconductor, and simultaneously improving the organic‐semiconductor morphology both on the contact surface and along the edges of the contacts.^[^ ^74^ ^]^

**Figure 5:**
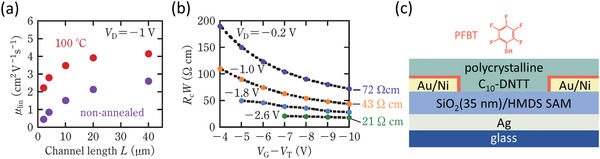
Low contact resistance achieved in bottom‐gate, bottom‐contact organic TFTs through implementation of a self‐assembled monolayer (SAM) of pentafluorobenzenethiol (PFBT) on the gold contacts and annealing of the transistors at a temperature of 100 °C in nitrogen to enhance the charge‐carrier mobility. a) Effective carrier mobility measured in the linear regime (*µ*_lin_) plotted as a function of the channel length. b) Width‐normalized contact resistance (*R*_C_ *W*) determined by TLM as a function of the gate overdrive voltage. Adapted with permission.^[30]^ Copyright 2017, The Japan Society of Applied Physics. c) Schematic cross‐section of the TFTs.

Since its first demonstration in organic transistors in 2005,^[^ ^160^ ^]^ pentafluorobenzenethiol (PFBT) has become by far the most widely utilized and effective thiol molecule to date for improving the hole injection in p‐channel organic TFTs.^[^ ^30^, ^33^, ^34^, ^46^, ^82^, ^155^, ^156^, ^157^ ^]^ In many ways, PFBT provides an ideal SAM for contact modification to improve the injection of holes in p‐channel TFTs, because it forms stable, well‐ordered monolayers^[^ ^158^ ^]^ with a large collective downward dipole moment^[^ ^161^ ^]^ and promotes the formation of an organic‐semiconductor thin‐film morphology with excellent in‐plane π‐stacking and a high charge‐carrier mobility.^[^ ^33^, ^82^, ^162^ ^]^ The aptitude of PFBT SAMs on gold contacts for enabling *R*_C_ *W* below 100 Ωcm was first demonstrated in 2017 by Kitamura (**Figure** **5**).^[^ ^30^ ^]^ In this case, PFBT‐treated gold contacts in bottom‐gate, bottom‐contact TFTs were combined with a vacuum‐deposited C_10_‐DNTT layer with enhanced charge‐carrier mobility achieved through postdeposition annealing at a temperature of 100 °C in nitrogen to remove defects in the crystal packing (Figure 5a).^[^ ^163^ ^]^ This led to a lowest *R*_C_ *W* of 72 Ωcm at *V*_GS_–*V*_th_ = −10 V from the TLM analysis performed at the lowest *V*_DS_ (−0.2 V; Figure 5b).

### 3.5 Low‐Dimensional Liquid Crystals of Small‐Molecule Semiconductors

**Figure 6:**
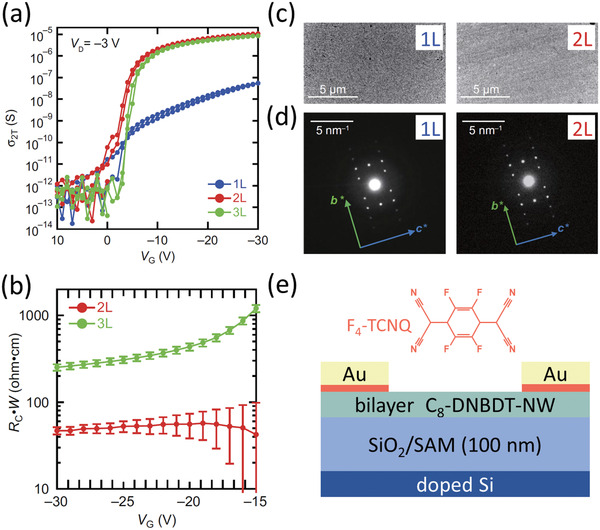
a) Two‐terminal conductivity (σ_2T_) as a function of the gate–source voltage measured on bottom‐gate, top‐contact organic TFTs based on monolayer (1L), bilayer (2L), and trilayer (3L) single crystals of the small‐molecule semiconductor C_8_‐DNBDT‐NW. b) Width‐normalized contact resistance as a function of the gate–source voltage for the bilayer and trilayer TFTs. c) TEM images and d) small‐angle electron diffraction patterns of the 1L and 2L crystals of C_8_‐DNBDT‐NW. Adapted with permission.^[31]^ Copyright 2018, American Association of the Advancement of Science. e) Schematic cross‐section of the TFTs that showed the lowest contact resistance. A thin layer of thermally evaporated F_4_‐TCNQ was implemented as a contact dopant.

In the years following 2017, at least one report of *R*_C_ *W* < 100 Ωcm has been published by a few different research groups each year. These results have been driven primarily by the implementation of high‐performing functional derivatives of DNTT and other highly π‐extended small‐molecule semiconductors^[^ ^164^ ^]^ in combination with optimized fabrication methods. In particular, the liquid‐crystalline properties of these and other organic semiconductors have seen rapid development in recent years.^[^ ^165^, ^166^, ^167^ ^]^ Owing to enhanced control of the crystallinity and morphology through various means, such as annealing and directional solution‐shearing, liquid‐crystalline organic semiconductors have shown the potential to realize charge‐carrier mobilities in excess of 10 cm^2^ V^−1^ s^−1^ in extremely thin films that can be fabricated into large‐area single crystals by solution processing.^[^ ^167^ ^]^ The potential for realizing additional improvements to the contact resistance by using liquid crystals was unequivocally demonstrated in a seminal paper by the Takeya group in 2018.^[^ ^31^ ^]^ The team compared bottom‐gate, top‐contact TFTs based on monolayer (1L), bilayer (2L), and trilayer (3L) crystals of 3,11‐dioctyldinaphtho[2,3‐*d*:2′,3′‐*d*′]benzo[1,2‐*b*:4,5‐*b*′]dithiophene (C_8_‐DNBDT‐NW) and a thin contact‐doping layer of 2,3,5,6‐tetrafluoro‐7,7,8,8,‐tetracyanoquinodimethane (F_4_‐TCNQ) (**Figure** **6**). While it might have been expected that the lowest contact resistance would be obtained for the monolayer crystals (since in staggered TFTs this would reduce the access resistance to a practical minimum by positioning the contacts as close as possible to the channel^[^ ^168^ ^]^), the lowest contact resistance (*R*_C_ *W* = 47 Ωcm) was actually demonstrated in the bilayer TFTs (Figure 6b). This was attributed to a smaller density of defects in the crystal packing compared to the monolayer crystals, as evidenced by atomic force microscopy (AFM) and small‐angle electron diffraction measurements (Figure 6c,d). Two follow‐up publications by the same group using similar devices have reported comparably low contact resistances,^[^ ^36^, ^37^ ^]^ leading to a concomitant enhancement in the on/off current ratio (up to 10^10^) and the transit frequency (up to 45 MHz).^[^ ^36^ ^]^

**Figure 7:**
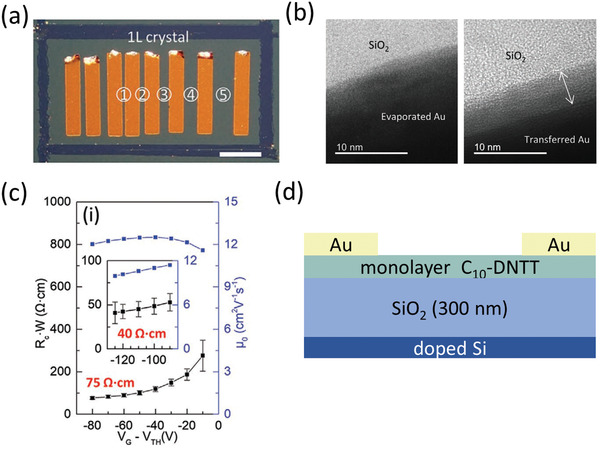
a) Photograph of bottom‐gate, top‐contact organic TFTs formed by placing gold contacts by mechanical transfer onto the surface of a C_10_‐DNTT monolayer crystal. b) Cross‐sectional TEM images of the interfaces between the SiO_2_ gate dielectric, the C_10_‐DNTT monolayer, and gold contacts deposited by thermal evaporation or mechanical transfer. c) Width‐normalized contact resistance and intrinsic channel mobility as a function of the gate overdrive voltage determined using TLM. Adapted with permission.^[^ ^35^ ^]^ Copyright 2020, Wiley‐VCH. d) Schematic cross‐section of the TFTs.

While analyses of the crystal structure suggested that the monolayer C_8_‐DNBDT‐NW crystals contained a higher density of packing defects that limited the TFT performance compared to the multilayer crystals, an additional factor that was not discussed is that the uppermost layer of the crystals may have degraded due to thermal damage during the deposition of the gold contacts (by thermal evaporation in vacuum).^[^ ^61^ ^]^ To combat this potentially critical effect, the Chan group implemented mechanically transferred gold contacts gently placed onto large‐area monolayer crystals of C_10_‐DNTT (**Figure** **7**a).^[^ ^35^ ^]^ Indeed, comparison of transmission electron microscopy (TEM) cross‐sections showed evidence of a sharper interface to the monolayer crystal with the transferred contacts compared to contacts deposited by thermal evaporation (Figure 7b), and TLM showed a low minimum *R*_C_ *W* = 40 Ωcm (Figure 7d). Of all organic transistors covered in this review, these devices share perhaps the greatest degree of similarity with FETs based on 2D inorganic semiconductors, especially TMDs.^[^ ^40^, ^41^ ^]^ This includes the use of mechanically transferred contacts, which is an approach that has also been implemented in 2D TMD transistors to form so‐called “van der Waals contacts” characterized by atomically sharp interfaces without chemically or kinetically damaging the 2D semiconductor.^[^ ^106^, ^169^ ^]^ The necessity of this and some other elaborate approaches for forming contacts to TMDs arises because substitutional doping is difficult to effectively implement or in many cases detrimental to the transistor characteristics including the contact resistance.^[^ ^41^ ^]^ This parallels the limitations of doping the contact regions in organic crystals and polycrystalline films, where the dopants may disrupt the molecular order and severely degrade the in‐plane charge‐carrier transport^[^ ^129^ ^]^ or poorly intercalate the crystal domains.^[^ ^139^ ^]^

### 3.6 Low‐Voltage Organic TFTs Using a Thin Gate Dielectric

**Figure 8:**
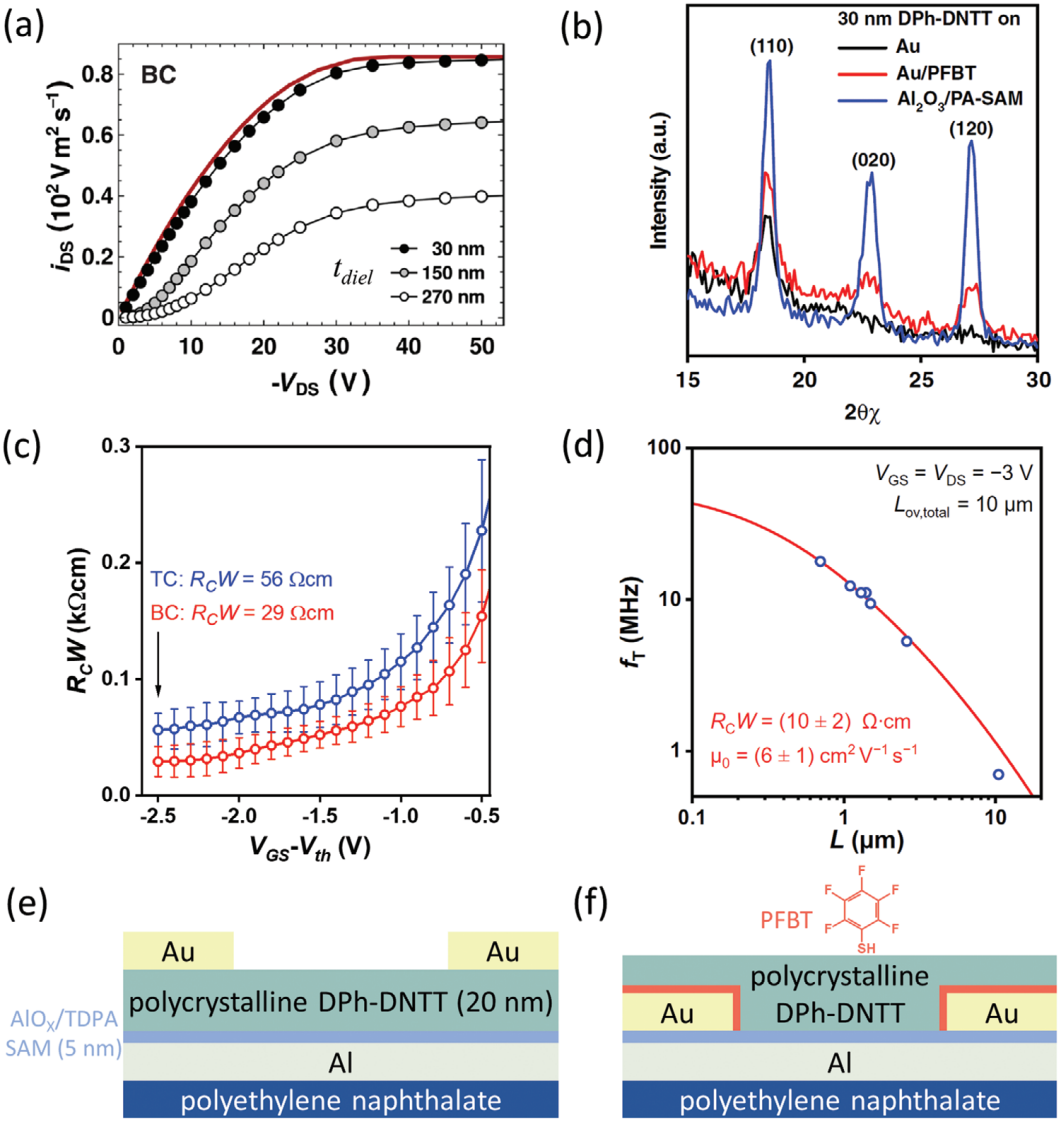
a) Simulated normalized output curves of bottom‐contact (BC) organic TFTs as a function of the gate‐dielectric thickness (*t*_diel_). Adapted with permission.^[^ ^83^ ^]^ Copyright 2015, American Physical Society. b) Grazing‐incidence X‐ray diffraction measurements on 30 nm thick DPh‐DNTT films on surfaces consisting of Au (black), PFBT‐treated Au (red), and atomic‐layer‐deposited Al_2_O_3_ passivated with an *n*‐tetradecylphosphonic acid (TDPA) SAM (blue). The (110), (020), and (120) diffraction peaks are clearly distinguished in both of the two latter cases, signifying strong in‐plane π–π stacking, while on bare Au, only the (110) peak is present. c) Width‐normalized contact resistance (*R*_C_ *W*) as a function of the gate overdrive voltage of top‐contact (TC) and BC TFTs. Adapted with permission.^[^ ^33^ ^]^ Copyright 2019, Springer Nature. d) Transit frequency (*f*_T_) as a function of the channel length (*L*) of BC TFTs. Adapted with permission.^[^ ^34^ ^]^ Copyright 2020, American Association for the Advancement of Science. e) Schematic cross‐section of the TC TFTs. f) Schematic cross‐section of the BC TFTs.

The final examples of low contact resistance (**Figure** **8**), including the standing record for organic TFTs of 10 Ωcm reported in 2020,^[^ ^34^ ^]^ were a result of several combined advantageous factors, but notably did not rely on the use of low‐dimensional crystals as in the previous section. The first key factor to achieving low contact resistance was a robust approach for making a very thin gate dielectric that enables low‐voltage operation and high‐mobility thin films of small‐molecule semiconductors.^[^ ^170^ ^]^ The importance of using a thin gate dielectric as it relates to contact resistance became apparent through a re‐evaluation of the effects of the device architecture (coplanar vs staggered) on the charge injection in organic TFTs.^[^ ^33^ ^]^ This was motivated by previous theoretical studies^[^ ^68^, ^83^ ^]^ in which it was shown through drift‐diffusion‐based simulations of organic TFTs that if the Schottky barrier is sufficiently minimized and the gate‐dielectric thickness is reduced, charge injection can become more efficient in coplanar TFTs, to the extent that a smaller contact resistance is achievable than in otherwise comparable staggered devices (Figure 8a). The second most important factor was the implementation of high‐quality thin films of small‐molecule semiconductors that can show high intrinsic hole mobilities.^[^ ^171^ ^]^ In particular, C_10_‐DNTT and 2,9‐diphenyl‐dinaphtho[2,3‐*b*:2′,3′‐*f*]thieno[3,2‐*b*]thiophene (DPh‐DNTT) were chosen based on previous demonstrations of low contact resistance in similarly fabricated TFTs based on these molecules.^[^ ^137^ ^]^ Finally, in the BC TFTs that ultimately showed the lowest contact resistances (29^[^ ^33^ ^]^ and 10 Ωcm^[^ ^34^ ^]^ for DPh‐DNTT and 31 Ωcm^[^ ^34^ ^]^ for C_10_‐DNTT), a SAM of PFBT was implemented on the gold source and drain contacts. The effects of PFBT on the DPh‐DNTT morphology are readily apparent from comparing grazing‐incidence X‐ray diffraction (GIXRD) measurements of DPh‐DNTT films deposited onto bare Au, PFBT‐treated Au, and the gate‐dielectric surface (Figure 8b). On PFBT‐treated Au, the (110), (020), and (120) diffraction peaks are clearly distinguished, indicating in‐plane π–π stacking in the DPh‐DNTT film,^[^ ^172^ ^]^ similar to the measurement of DPh‐DNTT on the optimized gate dielectric (Al_2_O_3_ passivated with *n*‐tetradecylphosphonic acid, TDPA). By contrast, a clear degradation of the in‐plane π‐stacking is observed for the bare Au surface.

It is also intriguing to note that in this study, even the TC TFTs showed exceptionally low *R*_C_ *W* = 56 Ωcm (Figure 8c). Perhaps most significant is that this was demonstrated without the use of contact dopants or other interfacial layers of any kind, using gold contacts deposited by thermal evaporation directly onto a relatively thick (20 nm) polycrystalline film of DPh‐DNTT. This potentially indicates that the vertical access resistance component from the vacuum‐deposited polycrystalline DPh‐DNTT thin‐films is exceptionally low in comparison to thin‐films of other organic semiconductors, making DPh‐DNTT potentially relevant also for vertical transistors, such as OPBTs.^[^ ^7^, ^47^ ^]^

## 4 Present Challenges for Realizing a Contact Resistance of 1 Ωcm or Lower in Organic TFTs

From the empirical evidence reviewed in the previous section, we will now try to elucidate a general approach for obtaining state‐of‐the‐art contact resistances in organic TFTs in either the coplanar or the staggered device architecture. The most critical factor regardless of the device architecture is undoubtedly to minimize the access‐resistance component of the contact resistance. This is primarily achieved through control of the morphology of the organic semiconductor to minimize structural defects affecting charge transport both near the interfaces with the contacts and within the channel region close to the contacts. This is essential for promoting the most efficient charge injection and transport and must be considered as a prerequisite for future studies seeking to develop new methods to reduce the contact resistance. For staggered TFTs, this often includes an additional requirement that the thickness of the organic semiconductor be as small as possible. Furthermore, comparing the evidence from the above reports suggests that the myriad methods for fine‐tuning other aspects of the interface, such as the contact work function and charge‐carrier density, have played a relatively secondary role in achieving the state‐of‐the‐art thus far.

To make an assessment about what the primary limiting factors are once the organic‐semiconductor morphology is sufficiently controlled, it should first be acknowledged that even for some of the above‐discussed organic transistors, which have nearly “ideal” characteristics (including low‐dimensional highly crystalline semiconductor films, high carrier mobilities, sharp contact interfaces, thin gate dielectrics), the contact resistance is still no better than about 10 Ωcm and thus still substantially higher than in state‐of‐the‐art inorganic transistors.^[^ ^40^, ^41^, ^42^ ^]^ This hints that there is a common limitation that has so far not been fully addressed in these devices which prevents the contact resistance from being reduced much further below 10 Ωcm. A compelling explanation for this observation is that the Schottky‐barrier height has not been sufficiently reduced by the various methods that have so far been implemented to improve the interface energetics, such as using strongly dipolar SAMs.^[^ ^77^, ^149^, ^161^ ^]^ The inability to tune the Schottky‐barrier height can likely be attributed to the evidently ubiquitous occurrence of FLP in the contacts to organic semiconductors.^[^ ^55^, ^56^, ^173^ ^]^ The likeliness of this explanation is supported by experimental and theoretical work both from within the organic‐semiconductor community and the broader transistor community, especially with respect to 2D semiconductors.^[^ ^40^, ^41^, ^42^ ^]^

In the next section, the origins of FLP in metal–semiconductor contacts in general are reviewed, and further unique aspects related to organic semiconductors are expounded upon. Finally, the possible solutions to overcoming the effects of FLP to enable lower contact resistance are explored.

### 4.1 Fermi‐Level Pinning at Metal–Organic Semiconductor Interfaces

Since the equilibrium position of the Fermi level (*E*_F_) in a metal–semiconductor contact depends most strongly on the work function of the contact material (φ_C_), this is often a major focal point of efforts to tune the interface energetics to enable efficient injection of holes or electrons into the semiconductor.^[^ ^8^ ^]^ The heights of the hole‐injection barrier (*Φ*_B,p_) and electron‐injection barrier (*Φ*_B,n_) are taken to be the energy difference between the respective transport levels (HOMO and LUMO) and *E*_F_ (Figure 1b). Many reports have demonstrated that using a different contact metal^[^ ^64^ ^]^ or otherwise modifying φ_C_ through chemical means like dipolar SAMs^[^ ^117^, ^151^ ^]^ can improve the contact resistance in organic transistors, ostensibly due to a reduction in the relevant injection‐barrier height. This is at least part of the explanation that is given for the often‐excellent contact resistance improvement of p‐channel bottom‐contact organic TFTs, which utilized PFBT and other fluorinated SAMs,^[^ ^154^, ^174^ ^]^ including the experimental instances reviewed in the previous section.^[^ ^30^, ^33^, ^34^ ^]^ This has thus motivated investigations into other thiol molecules capable of forming dipolar SAMs on the metal contacts with some particular emphasis on those with a larger number of fluorine atoms to induce a work‐function shift to higher energies beyond that obtained with PFBT.^[^ ^153^, ^154^ ^]^ However, the efficacy of these approaches relies on adherence to the so‐called Schottky–Mott limit, wherein *Φ*_B,p_ and *Φ*_B,n_ scale uniformly with respect to φ_C_ according to

(3) $$S=d\Phi_{B,n}\text{/d}\varphi_{c}=-d\Phi_{B,p}\text{/d}\varphi_{c}=1$$

While *S* = 1 can be observed in many cases for values of φ_C_ that fall within the bandgap of the semiconductor,^[^ ^55^, ^173^ ^]^ *S* can also be significantly smaller than unity and may gradually or abruptly decrease to zero at low and/or high φ_C_. The deviation of *S* from the Schottky–Mott limit is known generally as FLP and has many potential physical origins.^[^ ^55^, ^57^, ^107^, ^109^, ^175^, ^176^, ^177^ ^]^ There is growing evidence that the benefits of dipolar SAMs and similar approaches are inherently limited by FLP.^[^ ^77^, ^178^ ^]^

**Figure 9:**
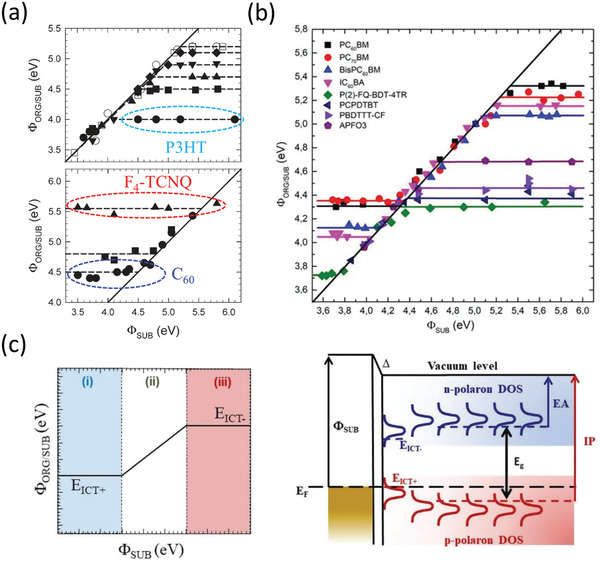
a) Survey of results from ultraviolet photoelectron spectroscopy (UPS) studies of various small‐molecule and polymeric semiconductors showing the dependence of the work function of the organic semiconductors (Φ_ORG_) on the substrate work function (Φ_SUB_). Data points for a few semiconductors relevant for organic transistors are highlighted. Adapted with permission.^[^ ^55^ ^]^ Copyright 2009, Wiley‐VCH. b) Survey of additional organic semiconductors of particular interest for the development of organic solar cells. In both panels (a) and (b), strong Fermi‐level pinning is evident from the sharply decreased scaling factors at low and high Φ_SUB_. c) Schematic diagrams outlining the integer charge‐transfer (ICT) explanation for FLP. Adapted with permission.^[^ ^173^ ^]^ Copyright 2019, John Wiley & Sons.

FLP in metal–organic semiconductor interfaces has been most prominently observed and studied through investigations using UPS.^[^ ^109^ ^]^ UPS is a powerful method that can be used to evaluate the interface energetics by measuring the semiconductor work function (*Φ*_ORG_) and its dependence on the substrate work function (*Φ*_SUB_) (**Figure** **9**).^[^ ^23^ ^]^ In this way, deviations from the Schottky–Mott limit at high or low substrate work functions have been observed for many organic semiconductors, including small‐molecule semiconductors and polymers (Figure 9a,b).^[^ ^55^, ^173^ ^]^

**Figure 10:**
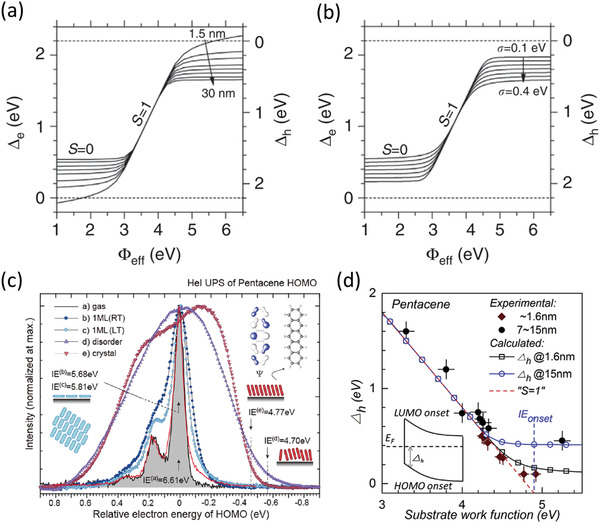
a) Simulations of the energy‐level offsets of pentacene showing the effects of a broader density of states via the standard deviation (σ_L_) for the LUMO on the Fermi‐level‐pinning (FLP) behavior at low substrate work functions. b) Simulations showing the effects of different pentacene film thicknesses on the FLP behavior indicated by reductions in *S* at the extreme ends of the effective work function. Adapted with permission.^[^ ^57^ ^]^ Copyright 2014, Springer Nature. c) High‐resolution ultraviolet photoelectron spectroscopy (UPS) measurements of the HOMO of pentacene in various states. In general, horizontally oriented monolayers (ML) show a much sharper density of states (DOS) comparable to the gas phase than “upright” oriented monolayer crystals or disordered layers, which are more relevant for thin‐film transistors. Adapted with permission.^[^ ^58^ ^]^ Copyright 2017, IOP Publishing. d) Analysis of the barrier height for holes (Δ_h_) of pentacene thin films as a function of the work function of the substrate. Using a film with smaller thickness (down to 1.6 nm) shows a significant reduction in the pinning level. Adapted with permission.^[^ ^56^ ^]^ Copyright 2017, Elsevier.

FLP has also been known for many years from studies of metal contacts to inorganic semiconductors, of which GaAs is a notorious example. In this case, the Fermi level can become pinned due to the formation of interface states with energies within the bandgap that occur due to adatom‐induced defects, metal‐induced gap states, chemical impurities, and/or crystallographic dislocations at the surface of the GaAs.^[^ ^179^ ^]^ While some of these explanations are applicable to metal–organic semiconductor contacts as well, the precise mechanisms for the occurrence of FLP in organic semiconductors are somewhat different and still under active investigation, given that the experimental evidence can support multiple nuanced interpretations.^[^ ^55^, ^56^, ^57^, ^58^ ^]^ One of the most prominent explanations for the occurrence of FLP at metal–organic semiconductor interfaces is that interfacial polarons, which are created through the spontaneous equilibration process across the interface lead also to integer charge‐transfer (ICT) states in the organic semiconductor with fixed energy levels for electrons (*E*_ICT−_) or holes (*E*_ICT+_) that are lower (higher) in energy than the bulk EA (IE) (Figure 9c).^[^ ^23^, ^110^ ^]^ Charge transfer between these states then results in an additional contribution to the interface dipole that leads to FLP. However, recent electrostatic modeling efforts (**Figure** **10**a,b)^[^ ^57^ ^]^ have shown that the presence of an ICT state is insufficient to account for the large minimum injection‐barrier heights that are often observed at interfaces that show FLP, such as Au–pentacene (Φ_B,p_ = 0.4 eV in reference ^[^ ^180^ ^]^). Experimental FLP results for several systems, including the dependence on film thickness, could potentially more generally be explained by the intrinsically broad DOS of the HOMO/LUMO levels that is a ubiquitous characteristic of organic semiconductors (Figure 10c).^[^ ^56^, ^176^ ^]^ Furthermore, the universality of this explanation extends beyond potential effects related to disorder at the interface and was expounded upon by Yang et al.^[^ ^56^, ^58^ ^]^ It was found that while the common assertion that the presence of a separate density of gap states in the bandgap of the semiconductor due to disorder can indeed contribute to FLP, it is not a prerequisite. Instead, the occurrence of FLP and by extension the magnitude of the minimum heights of the injection barriers that are possible for a given contact–organic semiconductor system could be predicted based entirely on the standard deviations of the assumed Gaussian HOMO and LUMO distributions in the organic semiconductor in weak contact with a metal (Figure 10d).^[^ ^56^ ^]^

The dependence of FLP on the broadness of the DOS of the LUMO or HOMO provides an implication that the route toward lower injection barriers and thus lower contact resistances in organic transistors coincides with narrowing the DOS. Indeed, this agrees well with the relative prominence of low‐dimensional liquid‐crystalline films of organic semiconductors in the above survey of the lowest contact resistances^[^ ^31^, ^35^, ^36^, ^37^ ^]^ and aligns with the general motivation for fabricating 2D organic crystals for transistors.^[^ ^167^, ^181^ ^]^ Fortunately, this may then put the elimination of FLP and thus realizing lower contact resistances in close alignment with the wider research interests in developing organic‐semiconductor thin films with higher intrinsic charge‐carrier mobilities, since this generally coincides with a narrower DOS.^[^ ^182^, ^183^ ^]^ While promising, more efforts are clearly needed to enable transistors with lower contact resistance using other promising highly crystalline organic semiconductors, such as 2‐decyl‐7‐phenyl[1]‐benzothieno[3;2‐*b*][1]benzothiophene (Ph‐BTBT‐C_10_)^[^ ^168^, ^184^ ^]^ and rubrene.^[^ ^185^ ^]^ One such limitation that remains even in the case of a narrower DOS is that these transistors are predominantly implemented in bottom‐gate, top‐contact architectures where the access resistance can remain a major detriment due to generally lower out‐of‐plane charge‐carrier mobilities.^[^ ^51^, ^168^ ^]^ Therefore, the development of an effective method for fabricating transistors based on low‐dimensional single crystals in a coplanar architecture may help to alleviate this disadvantage and take full advantage of the enhanced charge‐carrier mobility to reduce the contact resistance.^[^ ^186^ ^]^ The primary difficulty for such an approach is to “match” the surface‐energy characteristics of the contacts and the gate insulator using, e.g., SAMs^[^ ^187^ ^]^ to avoid disruption of the crystallization of the organic semiconductor across the edges of the contacts.

### 4.2 Depinning the Fermi Level at Metal–Organic Semiconductor Interfaces

**Figure 11:**
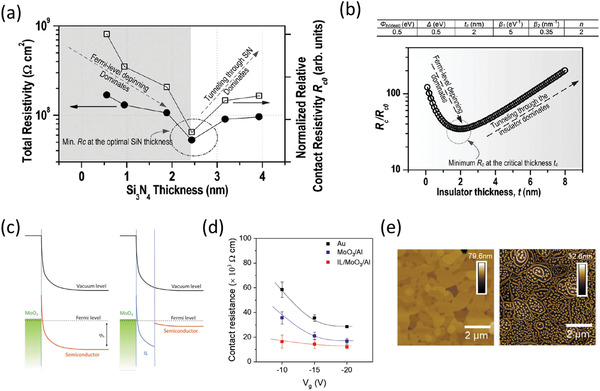
a) Measurements of the total and normalized relative contact resistivity in gold contacts to pentacene including a Si_3_N_4_ interlayer. b) Simulations evaluating the relative improvement in the contact resistance with the insulating layer thickness. Adapted with permission.^[^ ^190^ ^]^ Copyright 2010, American Physical Society. c) Schematic energy diagrams showing the effect of adding an interlayer (IL) with deep‐lying LUMO to the metal–organic semiconductor contact. d) Contact resistance as a function of the gate–source voltage in bottom‐gate, top‐contact organic transistors based on polycrystalline C_8_‐BTBT. Improvement in the contact resistance can clearly be seen by using MoO_3_ and an IL. e) Atomic force microscopy (AFM) height scans of the C_8_‐BTBT thin films without (left) and with (right) the IL deposited on top. Adapted with permission.^[^ ^139^ ^]^ Copyright 2020, John Wiley & Sons.

Besides the potential to eliminate FLP through narrowing the DOS, forming a more ideal metal–organic semiconductor interface and decoupling other sources of pinning, such as interface traps, might be achieved by inserting a thin interlayer. Such an approach has been shown to be effective for “depinning” the Fermi level in inorganic devices, e.g., using graphene or hexagonal boron nitride (hBN) interlayers to make contacts in 2D transistors based on TMDs,^[^ ^41^, ^106^ ^]^ and has to some extent been applied to organic transistors (**Figure** **11**).^[^ ^188^, ^189^ ^]^ Quasi‐Ohmic and tunneling contacts were also demonstrated between pentacene and gold by sputtering a thin interlayer (≈6 nm) of silicon nitride onto the pentacene film prior to depositing the gold contacts (Figure 11a).^[^ ^190^ ^]^ The accompanying theoretical simulations predicted an ideal thickness for the interlayer based on the intrinsic pinning properties of the contact–organic semiconductor system to balance depinning and tunneling of carriers across the interlayer (Figure 11b). However, the results showed that even for the ideal silicon‐nitride thickness of about 2 nm, the contact resistance would still be significantly higher than in the absence of an energy barrier at the interface.

Alternatively, SAMs may provide a more versatile avenue for introducing an interlayer to depin the Fermi level. Along with the added advantage of being inherently self‐patterning, the chain length of the SAM interlayer can be modified to tune its thickness, thus minimizing the tunneling barrier between the metal and organic semiconductor.^[^ ^150^ ^]^ The potential for SAMs to optimize the interface energetics extends beyond tuning the interlayer thickness and the work function.^[^ ^117^ ^]^ For instance, Lindell et al.^[^ ^113^ ^]^ showed that the pinning level in F_4_‐TCNQ could be adjusted using alkanethiol SAMs with different tail groups (CH_3_, COH, COOH) on gold contacts. Thus, while work‐function tuning with SAMs is limited by FLP, choosing an appropriate tail group for the SAM to change the charge‐screening properties and induce a more‐preferable pinning‐energy level within the organic semiconductor may be possible. However, the precise mechanism behind the changes in pinning level with the different tail groups needs more detailed investigation.

Finally, akin to the development of contact heterostructures in OLEDs, the interlayer need not be composed of a single component material but rather a stack of materials selected based on their energetic properties. This was demonstrated in recent work by Blom and co‐workers^[^ ^138^, ^139^ ^]^ using a strategy with a combined interlayer of a high‐work‐function metal oxide (MoO_3_) and an organic layer with a deep IE to improve hole injection in organic devices (Figure 11c). While this strategy proved to be a promising route for drastically improving the injection into relatively amorphous organic semiconductors by orders of magnitude, the results of further application to high‐mobility polycrystalline layers of 2,7‐dioctyl[1]benzothieno[3,2‐*b*][1]benzothiophene (C_8_‐BTBT) showed less‐compelling results with a lowest *R*_C_ *W* ≈ 15 kΩcm(Figure 11d), significantly higher than what has been demonstrated using, e.g., contact doping.^[^ ^191^ ^]^ The explanation given by the authors was that the tendency of the interlayer to agglomerate at the edges of the molecular terraces in the C_8_‐BTBT film prevents sufficient tuning of the interface energetics (Figure 11e). This problem could potentially be solved by adopting a coplanar device architecture, since controlling the homogeneity of the interlayer will in principle not depend strongly on the organic‐semiconductor morphology.

### 4.3 Doping the Organic‐Semiconductor Host

Doping with strong acceptors (donors) has proven to be a powerful tool for enhancing hole (electron) transport and injection into organic semiconductors, primarily by increasing the mobile charge‐carrier density and helping to alleviate some of the effects of molecular disorder, such as by filling trap states in the host organic semiconductor.^[^ ^128^, ^129^, ^130^, ^131^, ^132^ ^]^ Contact doping has been shown to be especially effective for narrowing the space‐charge region as the primary mechanism leading to lower contact resistance in organic transistors.^[^ ^192^ ^]^ However, while contact dopants were also implemented in some of the above cases with *R*_C_ *W* < 100 Ωcm,^[^ ^29^, ^31^, ^36^, ^37^ ^]^ it is not actually clear if the presence of contact dopants was instrumental in achieving these results. A much larger portion of the contact resistance in these devices seems to have instead been eliminated by minimizing the access resistance through reduction of the organic‐semiconductor thickness (see, e.g., Figures 4b and 6a).^[^ ^29^, ^31^, ^35^, ^168^ ^]^ This is further supported when considering the other cases surveyed above where dopants are completely absent and use either the same organic semiconductors or similar fabrication approaches.^[^ ^30^, ^34^, ^35^ ^]^

**Figure 12:**
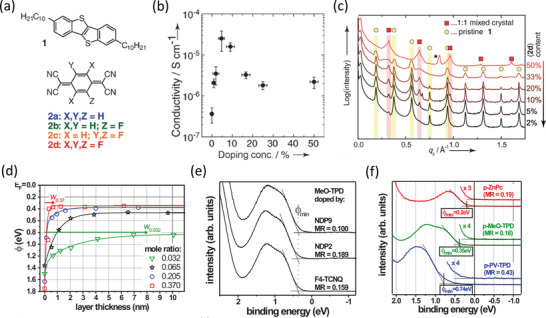
a) Prototypical example of a host–dopant system of C_8_‐BTBT and TCNQ with various degrees of fluorination. b) Conductivity as a function of doping concentration of C_8_‐BTBT doped with F_4_‐TCNQ. c) Specular X‐ray diffraction of C_8_‐BTBT doped with F_4_‐TCNQ. Higher concentrations result in a degradation of the crystal packing of the host (yellow highlighted peaks). Adapted with permission.^[^ ^129^ ^]^ Copyright 2013, Wiley‐VCH. d) Hole‐injection barrier (ϕ) as a function of layer thickness of MeO‐TPD doped with F_4_‐TCNQ. e) UPS spectra of the HOMO region in MeO‐TPD with three different dopants. f) UPS spectra of three different hosts doped with F_4_‐TCNQ. The hole‐injection barrier is dominated by the pinning level determined by the host. Adapted with permission.^[^ ^194^ ^]^ Copyright 2009, American Institute of Physics.

There are multiple possible explanations for this perceived lack of effectiveness of contact doping in the cases where the contact resistance is already below 100 Ωcm. To start, doping in organic semiconductors, especially in low‐dimensional crystals, has so far shown limited efficiency compared to doping inorganic semiconductors^[^ ^128^, ^129^, ^130^, ^193^ ^]^ or through ion penetration from an electrolyte as in EGOFETs and OECTs.^[^ ^28^, ^123^, ^124^, ^125^, ^126^, ^127^ ^]^ Often very high dopant concentrations are required to meaningfully enhance the conductivity of intrinsic organic‐semiconductor hosts primarily through trap filling, as shown, e.g., by Méndez et al. using F_4_‐TCNQ to dope C_8_‐BTBT (**Figure** **12**a–c).^[^ ^129^ ^]^ Furthermore, there is typically an upper limit to the doping concentration that can be added, since adding too many dopant molecules eventually proves to be detrimental to charge transport, due to disruption of the molecular packing of the host material and degradation of the transfer integrals (Figure 12c).^[^ ^128^, ^129^, ^130^, ^193^ ^]^ Some additional lack of efficiency may also be a result of the method by which contact doping is usually implemented in the fabrication process: In the above organic transistors that used contact doping showing low contact resistance^[^ ^29^, ^31^, ^36^, ^37^ ^]^ and in most other instances, it is common practice to add pure dopant molecules via thermal sublimation as an additional interlayer deposited after the organic‐semiconductor host and prior to the deposition of the contacts. This can potentially limit the efficient integration of dopants into the host‐semiconductor films where they could provide the greatest benefit for enabling efficient charge transfer. Furthermore, while dopants are clearly effective for reducing the width of the space‐charge region next to the metal contacts (Figure 12d), dopants may not be sufficient to counteract the limitations imposed by FLP. This was demonstrated by Olthof et al.^[^ ^194^ ^]^ using UPS measurements of various host films and dopants that showed that the Fermi level remains pinned to a fixed level determined by the host, regardless of the dopant strength and concentration (Figure 12c–e). Nonetheless, combining an efficient dopant to minimize the space‐charge region with an effective depinning strategy is a potentially promising route toward minimizing the effective injection barrier and thereby realizing lower contact resistances.

### 4.4 What Does an Ideal Contact Look Like?

During the writing of this report, an annealing‐free approach was introduced wherein the contact resistance in transistors based on TMDs could be almost completely eliminated by including a semimetal interlayer to form contacts free of metal‐induced gap states and with a Schottky barrier height reduced to zero.^[^ ^42^ ^]^ Using a semimetallic bismuth interlayer with MoS_2_ yielded an ultralow *R*_C_ *W* of 0.012 Ωcm, within an order of magnitude of the quantum limit over the measured charge‐carrier density range.^[^ ^40^ ^]^

Mastery over the contacts to TMDs did not happen overnight. Several challenges that share some analogue with the most‐advanced contacts to organic semiconductors had to be overcome through clever contact engineering.^[^ ^41^ ^]^ In particular,

– there typically is a van der Waals gap between the metal contact and the top‐most semiconductor layer (Figure 1e);
– substitutional doping is limited or impossible;
– out‐of‐plane mobility is much smaller than in‐plane mobility;
– FLP limits the degree to which the Schottky barrier can be reduced by work‐function tuning.

**Figure 13:**
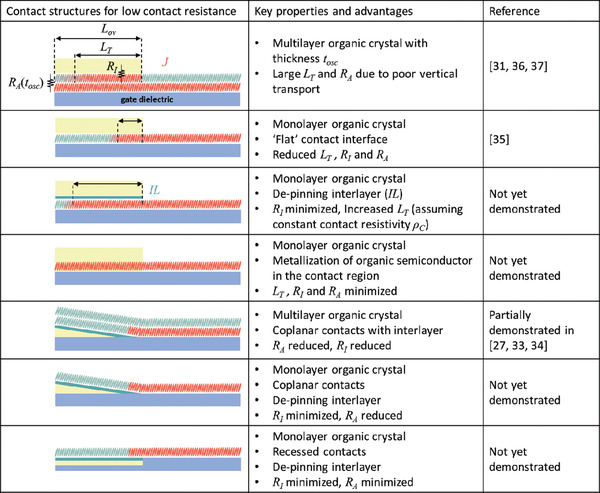
Contact idealization in organic transistors may take many forms. The multilayer organic crystal with a staggered architecture is limited by a larger access resistance (*R*_A_). The transfer length (*L*_T_) is indicative primarily of the sheet resistance under the contacts but can also be affected by the interface resistance (*R*_I_), with a larger *L*_T_ arising for a constant contact resistivity (ρ_C_). In addition, the contact overlap length (*L*_ov_) needs to be sufficiently larger than *L*_T_. In the coplanar architecture, an interlayer is necessary to control the semiconductor morphology; *R*_A_ is reduced and *R*_I_ dominates the contact resistance. Reduction to a monolayer crystal minimizes *R*_A_ and in combination with a depinning layer may lead to minimization of *R*_I_. Recessing the contacts into the gate insulator may yield additional improvement to the morphology transitioning from the contact to insulator surface. The potentially “ideal” case is that of a metallized organic layer in the contact region, which minimizes *L*_T_, *R*_I_, and *R*_A_. The “contact” will be formed by two phases of the same organic semiconductor (a highly conducting phase and the semiconducting phase).

The general unavailability of substitutional doping is the most critical limitation, as this constrains the degree to which the semiconductor itself can be modified to enhance the mobile charge‐carrier density. These issues have been partially circumvented in 2D transistors with a variety of methods, such as by forming so‐called edge contacts where the metal contact is fabricated such that charges are injected at the high‐mobility in‐plane edge of the 2D crystal.^[^ ^195^ ^]^ Since organic molecular crystals are formed by van der Waals interactions between molecules, edge contacts may not be feasible due to disturbances to the molecular‐packing structure upon contact formation.^[^ ^59^, ^61^ ^]^ Interlayers may therefore continue to play a role as they have in some TMD devices, such as MoTe_2_ transistors with scandium contacts, where a single‐atom‐thick layer of hexagonal boron nitride can be inserted to prevent FLP and the (in this case) detrimental reaction of the first few layers of MoTe_2_ with the scandium.^[^ ^196^ ^]^ In any case, for practical contacts where an out‐of‐plane contribution to charge injection is inevitable, a semihierarchical series of contact structures can be envisioned, following inspiration from the contacts to TMDs (**Figure** **13**). In Figure 13, a comparison is made between demonstrated low‐*R*_C_ structures in organic transistors and their potentially more “ideal” counterparts.

To achieve an ideal situation, the van der Waals gap would likely need to be minimized or eliminated entirely by hybridization of the metal with the first layer of molecules in the semiconductor to minimize this contribution to the injection barrier.^[^ ^41^, ^169^ ^]^ In the case of a single‐monolayer crystal, ideally the entire layer of metallized semiconductor underneath/above the metal contact would then act as the contact to the nonmetallized channel region. This can be achieved in TMD transistors by careful selection of the contact metal and the semiconductor with additional steps to remove surface contaminants.^[^ ^40^ ^]^ Potential drawbacks to be aware of are that hybridization of the metal with the TMD is not always beneficial,^[^ ^196^ ^]^ and this approach often requires subsequent high‐temperature annealing processes to complete the reaction at the interface (this of course excepts the recent development discussed at the start of this section^[^ ^42^ ^]^). For organic transistors, a low‐temperature process would need to be implemented to form similarly intimate contact interfaces, so as not to destroy the semiconductor and to maintain applicability to flexible substrates.

The development of low‐temperature solutions for forming more intimate interfaces to organic semiconductors might directly benefit from the collective research efforts on molecular electronic junctions^[^ ^197^ ^]^ and hybridized‐molecular monolayers on metals.^[^ ^24^, ^100^ ^]^ In particular, efforts to understand the role of anchoring groups on the efficiency of charge transfer from a metal contact to a chemisorbed molecule may prompt the design of more effective SAMs or organic semiconductors with prospects for direct hybridization with the metal contacts.^[^ ^198^ ^]^ It may also be possible to implement a metal–organic hybridization strategy with a donor (acceptor) molecule that forms a single‐atom‐thick chemisorbed layer on metal contacts to improve electron (hole) injection. Various studies have revealed methods for achieving such selective formation of strongly bonded molecular monolayers to metals, including F_4_‐TCNQ on gold^[^ ^199^ ^]^ and hexaazatriphenylene‐hexanitrile (HATCN)^[^ ^200^ ^]^ and phthalocyanines on silver,^[^ ^201^ ^]^ showing that hybridized states occur upon chemisorption to the metal surface. Reduction of the bandgap upon interfacing with a metal surface has also been commonly observed in some other aromatic molecules,^[^ ^105^, ^202^ ^]^ indicating the possibility to metalize the first layer of the semiconductor or the interlayer, but these approaches have to our knowledge not yet been applied to contacts in state‐of‐the‐art organic TFTs showing low contact resistance.

Finally, it should be reemphasized that the design of an “ideal” contact will likely remain largely holistic, with a “universal” strategy unlikely to ever be fully realized. In all of these strategies, care would have to be taken to mitigate detrimental effects on a case‐by‐case basis, such as the interface dipole introduced by, e.g., the push‐back effect arising from the van der Waals and Pauli exclusion interactions between the metal contact and the organic molecules.^[^ ^203^ ^]^ A persistent major challenge will also be attempting to implement a well‐ordered organic‐semiconductor layer across the contact‐to‐channel interface, which would presumably have different surface chemistries and reactivities (metals, oxides, or other organics). Further, a hybridized interface does not necessarily do anything to help with FLP, and the so‐far developed depinning approaches, such as the use of an insulating interlayer,^[^ ^190^ ^]^ are mutually exclusive in principle to the hybridization approaches for removing the van der Waals gap. In general, development of all of these approaches will likely be best guided by corresponding density‐functional‐theory calculations of interface layers on metals,^[^ ^204^ ^]^ in combination with experimental investigations focusing on the organic semiconductors that have already shown excellent contact resistance, such as DNTT and its derivatives, wherever possible.

## 5 Summary and Outlook

A variety of methods have been developed to improve the contact properties in organic transistors over the past 20+ years. However, large contact resistance continues to be a major source of nonideality in organic transistors and presents a severe limitation for applying organic transistors in widespread commercial electronics applications. For the few reported cases covered in this report that have shown width‐normalized contact resistances below 100 Ωcm, the most impactful parameter nearly to the exclusion of all others so far has been control of the intrinsic organic‐semiconductor morphology near the contacts. Other methods developed for improving the contact resistance, such as contact doping and modifying the energy‐level alignment of metal contacts to the charge‐transport levels, by contrast, have evidently played a comparatively limited role in achieving the state‐of‐the‐art so far. This hints that a limiting factor persists once the organic‐semiconductor morphology is sufficiently controlled and has yet to be addressed adequately by these other methods. A compelling explanation may be found in the evidently ubiquitous and obtrusive occurrence of Fermi‐level pinning in metal–organic semiconductor interfaces. Given that there is apparently a strong link between the appearance of Fermi‐level pinning and the generally broad density of states of the transport levels in condensed thin films of organic semiconductors, it can be predicted that molecular engineering of the organic‐semiconductor layer specifically to narrow the density of states is paramount for reducing the contact resistance beyond what is currently achievable in the state‐of‐the‐art systems discussed in this report. The significant recent strides in developing ultrathin monolayer crystals for organic transistors has shown some promise and will more than likely play a key role going forward. The essentiality of contact doping remains to be fully proven, but the development of optimized dopant–host systems that sufficiently reduce the width of the space‐charge region may enable further improvements by minimizing the need to reduce the height of the Schottky barrier, thus potentially circumventing the detrimental effects of Fermi‐level pinning. Chemical modification of the metal contacts using SAMs remains relevant, but the working principles must necessarily be extended beyond their effects on the work function of the contacts to include the detailed effects that the SAM chemistry has on the interfacing organic‐semiconductor layer. Beyond these more well‐known or proven methods, some potential avenues to improve the contact–organic semiconductor interface remain to be thoroughly explored, such as the use of interlayers to depin the Fermi level or methods to metallize the first interfacing layer(s) of the organic semiconductor. In any case, we emphasize that it would be beneficial going forward to require any new methods or claims of further progress in reducing the contact resistance to first unequivocally account for any potential effects related to changes in the organic‐semiconductor morphology, given its clear dominance on the charge‐injection characteristics.

## Conflict of Interest

The authors declare no conflict of interest.
